# Predictive value of lymphocytopenia and the neutrophil-lymphocyte count ratio for severe imported malaria

**DOI:** 10.1186/1475-2875-12-101

**Published:** 2013-03-18

**Authors:** Marlies E van Wolfswinkel, Klaske Vliegenthart-Jongbloed, Mariana de Mendonça Melo, Peter C Wever, Matthew B McCall, Rob Koelewijn, Jaap J van Hellemond, Perry J van Genderen

**Affiliations:** 1 Department of Internal Medicine, Harbour Hospital Rotterdam, Rotterdam, The Netherlands; 2 Department of Medical Microbiology and Infection Control, Jeroen Bosch Hospital, ’s-Hertogenbosch, The Netherlands; 3 Department of Medical Microbiology and Infectious Diseases, Erasmus MC, Rotterdam, The Netherlands; 4 Laboratory of Parasitology, Harbour Hospital and Institute for Tropical Diseases, Rotterdam, The Netherlands; 5 Institute for Tropical Diseases, Harbour Hospital Rotterdam, Haringvliet 72, Rotterdam, TG, 3011, The Netherlands; 6 Travel Clinic Havenziekenhuis, Rotterdam, The Netherlands

**Keywords:** Malaria, Severity, Falciparum, NLCR, Lymphocytopenia, Leukocyte count, Severe disease, Marker, Biomarker

## Abstract

**Background:**

Lymphocytopenia has frequently been described in patients with malaria, but studies on its association with disease severity have yielded conflicting results. The neutrophil/lymphocyte count ratio (NLCR) has been introduced as a parameter for systemic inflammation in critically ill patients and was found, together with lymphocytopenia, to be a better predictor of bacteraemia than routine parameters like C-reactive protein and total leukocyte count. In the present study, the predictive value of the NLCR and lymphocytopenia for severe disease was evaluated in patients with imported malaria.

**Methods:**

All patients diagnosed with malaria at the Harbour Hospital between January 1^st^ 1999 and January 1^st^ 2012 with differential white cell counts determined within the first 24 hours after admission were included in this retrospective study. Severe malaria was defined according to the WHO criteria. The performance of the NLCR and lymphocytopenia as a marker of severe malarial disease was compared back-to-back with that of C-reactive protein as a reference biomarker.

**Results:**

A total of 440 patients (severe falciparum malaria n = 61, non-severe falciparum malaria n = 259, non-falciparum malaria n=120) were included in the study. Lymphocytopenia was present in 52% of all patients and the median NLCR of all patients was 3.2. Total lymphocyte counts and NLCR did not differ significantly between groups. A significant correlation of total leukocyte count and NLCR, but not lymphocyte count, with parasitaemia was found. ROC analysis revealed a good negative predictive value but a poor positive predictive value of both lymphocytopenia and NLCR and performance was inferior to that of C-reactive protein. After complete parasite clearance a significant rise in total leukocyte count and lymphocyte count and a significant decrease in NLCR was observed.

**Conclusion:**

The NLCR was found to correlate with parasitaemia, but both lymphocytopenia and the NLCR were inferior to C-reactive protein as markers for severe disease in patients with imported malaria. The NLCR and lymphocytopenia are not useful as predictive markers for severe disease in imported malaria in the acute care setting.

## Background

Changes in blood cell counts are a well-known feature of malaria. Alterations in leukocyte counts are often less pronounced than in the other blood cell lineages, but in general total leukocyte counts have been found to be low to normal in malaria [1,2]. Lymphocytopenia has frequently been described in malaria patients in endemic areas [3-6], and was found to be present in 63% of patients with imported *Plasmodium falciparum* infection [7]. Studies on the correlation between lymphocyte count and malaria severity yielded conflicting results, as both lymphocytopenia [1,3,4] and lymphocytosis [8] have been reported to be associated with adverse outcome.

Lymphocytopenia accompanied by a rise in neutrophil count is commonly seen in various infectious and non-infectious causes of systemic inflammation and stress [9-13]. Zahorec *et al.* introduced the ratio of neutrophil to lymphocyte count as a parameter of systemic inflammation and stress in critically ill surgical and medical patients [14]. The predictive value of both lymphocytopenia and the neutrophil-lymphocyte count ratio (NLCR) for bacteraemia was confirmed in a study in an emergency care setting, in which these parameters were found to be better predictors of bacteraemia than routine parameters like C-reactive protein (CRP) level, total leukocyte count or neutrophil count. Recently, another study evaluated this parameter in patients with a community-acquired pneumonia (CAP) [15] and it was found to predict severity and outcome of CAP with a higher prognostic accuracy as compared with traditional infection markers.

Although the NLCR is currently not routinely used as a clinical parameter, the above mentioned studies have demonstrated its value as an infection marker in critically ill patients. Unequivocal data concerning the predictive value of lymphocytopenia and the NLCR in malaria are not yet available.

In non-endemic countries, where malaria is only seen as an imported disease, non-specialized hospitals often rely on rapid diagnostic tests for the diagnosis of malaria and generally lack experience in the examination of thick and thin blood smears to asses the parasite load. There is, therefore, still a need for simple and readily available parameters for the early identification of patients at risk of severe or complicated disease. The present study evaluated lymphocytopenia and the NLCR as predictive markers of severe disease in a large cohort of patients with imported malaria.

## Methods

### Patients

The Harbour Hospital is a 161-bed general hospital located in Rotterdam, The Netherlands. It also comprises the Institute of Tropical Diseases, which serves as a national referral centre. All patients diagnosed with malaria at our centre are included in the Rotterdam Malaria Cohort study. Demographic, clinical and laboratory data of all these patients are collected using a standardized form and stored in an electronic database. For the present observational cohort study, patients diagnosed with malaria between January 1^st^ 1999 and January 1^st^ 2012 and with differential white cell counts determined within the first 24 hours after admission were included.

### Laboratory investigations

Total and differential leukocyte counts were measured using an automatic cell counters. During the study period three distinct cell counters were subsequently used after careful calibration (Sysmex NE 8000 [in the period January 1st 1999 -July 31st 2002], Beckman Coulter HMX [July 31st 2002- July 31st 2010] and Sysmex XE 2100 [July 31st 2010 - January 1st 2012], respectively). Absolute numbers of lymphocytes and neutrophil subsets were obtained by multiplication of the absolute leukocyte counts with their respective differential leukocyte counts. Manual confirmation of automatic cell count results was performed when immature or aberrant leukocytes, erythrocytes or platelet clumps were detected and when cell count results differed substantially from normal values. Other available laboratory examinations included red blood cell counts, haematocrit, platelet counts, C-reactive protein levels, serum electrolytes, total bilirubin, serum creatinine and urea, sodium, potassium, liver enzymes, blood glucose and venous plasma lactate.

### Detection of *Plasmodium* parasites

The standard procedure to diagnose malaria comprised a Quantitative Buffy Coat (QBC) analysis, a rapid diagnostic test (RDT) for malaria antigens (Binax NOW® Malaria Test Binax, Inc. Maine, USA), and thick and thin blood smears using freshly collected blood specimens from finger pricks. The RDT and the QBC analysis were performed according to the manufacturer’s instructions. QBC capillaries were examined independently by two technicians by microscopic analysis of two complete rows of the region between the bottom of the capillary and the polynuclear leukocyte layer using an Olympus BX-60 fluorescence microscope equipped with UV-filter, 50x objective and 12.5x oculars (total magnification 625x). These two lanes represent about 100 microscopic fields (at 625 × magnification) and take an average examination time of 5 min. Subsequently, the polynuclear and mononuclear cell layer was screened for schizonts, gametocytes, malaria pigment and elderly trophozoites of *Plasmodium vivax* and *Plasmodium ovale*.

Thick blood smears were stained with Field’s stain (Brunschwig Chemie, Amsterdam, the Netherlands) and thin smears with Diff Quick stain (Medion Diagnostics, Düdingen, Switzerland). Both staining procedures have been optimized for optimal staining of *Plasmodium* parasites as well as Schüffner’s dots and Maurer’s clefts in infected erythrocytes. Thick and thin smears were examined with regular light microscopes at a total magnification of 1250x.

The same *Plasmodium* detection methods were used throughout the whole study period.

## Definitions

### Severe malaria

Patients were classified as having severe malaria if they met one or more of the WHO criteria for severe malaria, as published [16], either on presentation or later during hospital admission.

### Leukocyte counts and NLCR

Leukocytopenia was defined as a leukocyte count of less than 4.0 × 10^9^/L and leukocytosis was defined as a leukocyte count exceeding 10.0 × 10^9^/L. Lymphocytopenia was defined as a lymphocyte count of less than 1.0 × 10^9^/L and lymphocytosis as a lymphocyte count more than 4.0 × 10^9^/L. Neutropenia was defined as a neutrophil count of less than 1.5 × 10^9^/L and neutrophilia as a neutrophil count of more than 7.0 × 10^9^/L. The neutrophil-lymphocyte count ratio was defined as the ratio of the absolute neutrophil count to the absolute lymphocyte count.

### Estimation of immunity to *P. falciparum*

The degree of immunity to malaria was estimated as previously described [17]. Adult immigrants from a malaria-endemic country living in the Netherlands were considered partially immune, because they had likely been exposed to *P. falciparum* during childhood. Patients who had been living in a malaria-endemic area for at least 2 years at the time of diagnosis were presumed semi-immune. Tourists from non-endemic regions who travelled to endemic areas were considered non-immune.

### Statistical analysis

Data were not normally distributed (Kolmogorov-Smirnov test) and are therefore presented as medians and range. Univariate comparisons were performed using the Chi-Square test and the Kruskall-Wallis test with Dunn’s post-hoc tests (three groups), or the Mann–Whitney test and Fisher’s Exact test (two groups). Correlations with parasitaemia were calculated using Spearman’s rank correlation. For the comparisons of differential leukocyte counts before and after treatment, Wilcoxon’s matched pairs test was used.

For clinical reference, the diagnostic performance of NLCR and lymphocytopenia for severe disease was compared to that of the classic biomarker serum CRP using receiver operating characteristic (ROC) analysis. The optimal cut-off point was identified using Youden’s index. The areas under the ROC curve (AUROCs) were compared to that of CRP in a pair-wise comparison by the method of Hanley and McNeil [18].

### Ethical approval

Given its retrospective observational design, ethical approval of this study was not required, according to the Dutch Medical Research Involving Human Subjects Act.

## Results

### Patient characteristics

Between January 1^st^ 1999 and January 1^st^ 2012 a total of 562 cases of imported malaria were seen. Differential leukocyte counts were available for 440 (78%) of the patients. Of these cases, 120 cases were caused by non-falciparum *Plasmodium* species: 88 by *P. vivax*, 27 by *P. ovale*, four by *Plasmodium malariae* and one by *Plasmodium knowlesi*. The majority of infections (320 or 72%) was caused by *P. falciparum,* including three patients who had a mixed infection; two with *P. falciparum* and *P. ovale*, one with *P. falciparum* and *P. vivax*. Sixty-one patients were classified as having severe malaria. All these patients met the severity criteria upon admission to the hospital, and no patients were re-classified as having severe malaria because of progression of disease during admission. Concomitant infection was present in 29 (7%) of patients and was more common in patients with severe malaria. None of the patients had positive blood cultures. The general characteristics of these patients are shown in Table 1.

**Table 1:** General characteristics

	**Severe P. falciparum**	**Non-severe P. falciparum**	**Non-falciparum**	**P value**
	**n=61**	**n=259**	**n=120**	
**Demographics**				
Age, years	44 (4–70)	39 (11–78)	36 (15–77)	0.0005^*^
Male/female, n (%)	39 (64%)/ 22 (36%)	191 (74%) / 68 (26%)	83 (69%)/37 (31%)	NS^#^
**Immunity to *P. falciparum***				0.0029^#^
**Non-immune**	42 (69%)	123 (47%)	N/A	
**Partially immune**	19 (31%)	118 (46%)	N/A	
**Semi-immune**	0 (0%)	3 (1%)	N/A	
**Unknown**	0 (0%)	16 (6%)	N/A	
**Continent of acquisition**				NS^#^
Africa, n (%)	56 (92%)	240 (93%)	42 (35%)	
Asia, n (%)	3 (5%)	11 (4%)	50 (42%)	
South and Central America, n (%)	1 (2%)	5 (2%)	26 (22%)	
Unknown, n (%)	1 (2%)	3 (1%)	2 (2%)	
**Duration of signs/symptoms**				0.007^#^
<8 days, n (%)	38 (62%)	177 (68%)	58 (48%)	
8-14 days, n (%)	17 (28%)	45 (17%)	24 (20%)	
15-28 days, n (%)	2 (3%)	20 (8%)	11 (9%)	
>28 days, n (%)	0 (0%)	5 (2%)	8 (7%)	
Unknown, n (%)	4 (7%)	12 (5%)	19 (16%)	
**Use of malaria chemoprophylaxis**				<0.0001^#^
No chemoprophylaxis, n (%)	49 (80%)	182 (70%)	51 (43%)	
Inadequate use, n (%)	8 (13%)	46 (18%)	15 (13%)	
Adequate use, n (%)	2 (3%)	20 (8%)	42 (35%)	
Unknown, n (%)	2 (3%)	11 (4%)	12 (10%)	
**Vital signs on admission**				
Body temperature, °C	38.4 (35.7-41.2)	38.5 (35.5-41.0)	38.9 (36.0-41.2)	NS*
Pulse rate, beats per minute	103 (50–150)	90 (45–140)	90 (58–138)	0.0005*
Systolic blood pressure, mm Hg	117 (80–160)	120 (73–185)	120 (90–196)	N.T.
Impaired consciousness (GCS<15), n (%)	8 (13%)	1 (0%)	0 (0%)	N.T.
Coma (GCS≤11), n (%)	3 (5%)	0 (0%)	0 (0%)	N.T.
**Laboratory data on admission**				
Haemoglobin, mmol/L	7.8 (2.5-10.9)	8.4 (4.0-11.1)	8.2 (4.6-11.2)	N.T.
Thrombocytes, × 10^9^/L	39 (3–188)	101 (18–293)	95 (10–292)	<0.0001*
C-reactive protein, mg/L	182 (65–476)	85 (5–320)	71 (14–348)	<0.0001*
Serum creatinine, μmol/L	114 (39–1081)	93 (47–238)	90 (53–255)	N.T.
Serum sodium, mmol/L	131 (115–146)	135 (119–145)	135 (124–148)	<0.0001*
Lactate dehydrogenase, U/L	485 (139–2038)	268 (118–947)	246 (127–775)	<0.0001*
Total bilirubin, μmol/L	53 (13–416)	22 (4–164)	22 (3–99)	N.T.
Plasma lactate, mmol/L	2.3 (0.6-6.2)	1.4 (0.5-4.6)	1.3 (0.6-4.0)	<0.0001*
**Leukocyte counts**				
Total leukocyte count, × 10^9^/L	6.9 (2.5-18.5)	4.9 (1.3-13.4)	5.3 (1.9-15.3)	<0.0001*
Neutrophil count, × 10^9^/L	3.6 (0.6-10.4)	3.1 (0.4-11.0)	3.2 (1.0-11.6)	0.0064*
Neutrophil count%	60 (19–85)	64 (13–91)	64 (20–88)	NS*
Lymphocyte count, × 10^9^/L	1.0 (0.2-4.2)	0.9 (0.1-5.0)	1.1 (0.3-6.1)	NS*
Lymphocyte count %	15 (4–45)	19 (2–74)	21 (5–74)	0.0036*
NLCR	3.5 (0.7-17.0)	3.3 (0.2-46)	2.8 (0.3-17.6)	NS*
**Parasite count**				
*P.falciparum* load (asexual parasites/μL)	230,000 (520–1,380,600)	4,288 (2–208,000)	N/A	N.T.
Presence of *P. falciparum* schizonts%)	33 (54%)	11 (4%)	N/A	<0.0001^
**Concomitant infection**				
All concomitant infections	8 (13%)	11 (4%)	10 (8%)	<0.0001^#^
Concomitant bacterial infection	5 (8%)	3 (1%)	2 (2%)	0.004^#^

* Comparison of 3 groups using the Kruskal Wallis test, ^#^ Comparison of 3 groups using the Chi-Square Test, ^ Comparison of 2 groups using Fisher’s Exact test. Parameters that are included in the modified WHO severity criteria for severe falciparum malaria were not tested (N.T.).

### Leukocyte counts and NLCR

**Figure 1:**
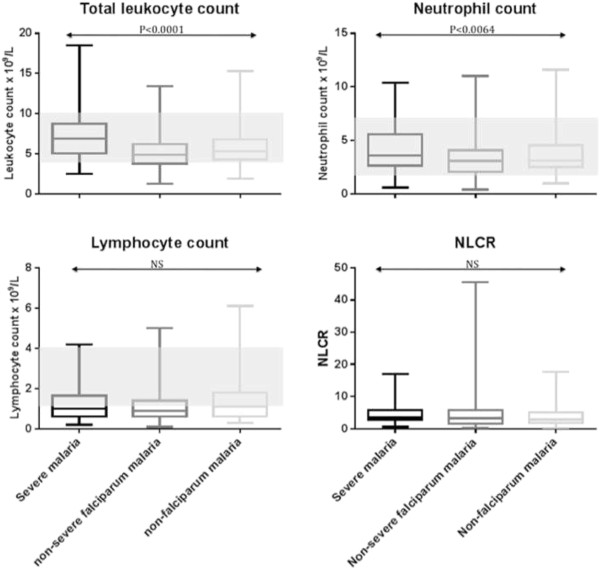
Distribution of leukocyte counts and the NLCR in patients with malaria grouped by severity and causative Plasmodium species.

Leukocytopenia was present in 23% (100/440) of all patients but in only 11% (7/61) of patients with severe malaria (Figure 1). Leukocytosis was seen in 4% (19/440) of all patients and was more common in patients with severe disease (13% or 8/61) compared to those with non-severe falciparum malaria (2% or 6/259) and non-falciparum malaria (4% or 5/120). Likewise, leukocyte counts were higher in patients with severe malaria compared to those with non-severe *P. falciparum* malaria and non-falciparum malaria (Table 1).

Lymphocytopenia was present in 52% (227/440) of all patients and was more frequently seen in patients with non-severe falciparum malaria (143/259 or 55%) than in patients with severe malaria (28/61 or 46%), and non-falciparum malaria (54/120 or 45%). Absolute lymphocyte counts did not differ significantly between the three groups.

Neutropenia was found in 8% (36/440) of all patients and neutrophilia in 4% (17/440), but was more common in patients with severe malaria (7% or 4/61) compared to those with non-severe falciparum malaria and non-falciparum malaria (3% or 7/259 and 5% or 6/120, respectively). Neutrophil counts were higher in patients with severe malaria compared to those with non-severe *P. falciparum* malaria and non-falciparum malaria (Table 1).

The median NLCR of all patients was 3.2. Although there was a trend towards higher values in patients with severe malaria (3.5) compared to those with non-severe *P. falciparum* (3.3) and non-falciparum (2.8) malaria, these differences were not significant.

### Correlation with parasitaemia

Significant correlations were found between parasitaemia and total leukocyte count (r_s_ 0.304, p<0.0001) and parasitaemia and NLCR (r_s_ 0.165, p=0.03).

Correlations between parasitaemia and absolute lymphocyte or neutrophil counts were not significant.

### Predictive value and ROC analysis

Analysis of the diagnostic performance of lymphocytopenia and the NLCR for the detection of severe malaria revealed good negative predictive value (0.87 and 0.92 respectively) but poor positive predictive value (Table 2). The AUROCs of total leukocyte count, neutrophil count, lymphocyte count and NLCR were all significantly inferior to that of CRP (Table 2).

**Table 2:** Descriptive statistics of diagnostic performance for severe malaria

**Parameter**	**Cut-off value**	**Sensitivity**	**Specificity**	**PPV**	**NPV**	**Youden’s**	**AUROC**
**Total leukocyte count**	≥ **6.5 × 10**^**9**^**/L**	0.59 (0.46-0.71)	0.75 (0.71-0.80)	0.28 (0.21-0.37)	0.92 (0.88-0.95)	0.34	0.70 (0.63-0.78)
**Neutrophil count**	≥ **3.4 × 10**^**9**^**/L**	0.64 (0.51-0.76)	0.56 (0.51-0.61)	0.19 (0.14-0.25)	0.91 (0.86-0.94)	0.2	0.61 (0.53-0.69)
**Lymphocyte count**	**< 0.7 × 10**^**9**^**/L**	0.33 (0.22-0.46)	0.72 (0.67-0.76)	0.16 (0.10-0.24)	0.87 (0.83-0.90)	0.05	0.51 (0.43-0.59)
**NLCR**	≥ **2.8**	0.77 (0.64-0.86)	0.44 (0.39-0.49)	0.18 (0.14-0.24)	0.92 (0.87-0.96)	0.21	0.57 (0.50-0.64)
**CRP**	**>141 mg/L**	0.80 (0.67-0.89)	0.76 (0.71-0.80)	0.33 (0.25-0.42)	0.96 (0.93-0.98)	0.56	0.84 (0.79-0.89)

### Leukocyte count changes during malaria treatment

Follow-up differential leukocyte counts were available for 40 (66%) patients with severe malaria and 114 patients with non-severe *P. falciparum* infection. By the time of complete parasite clearance (confirmed by a negative QBC and thick blood smear) a significant rise in total leukocyte count and lymphocyte count and a significant decrease in NLCR was observed. A significant decrease in neutrophil counts after treatment was only seen in non-severe malaria patients, but not in patients with severe malaria (Table 3).

**Table 3:** Leukocyte count changes during malaria treatment

	**On admission**	**Upon clearance of parasitaemia**	**n**	**P value**
**Severe *P. falciparum***				
CRP, mg/L	184 (108–373)	49 (1–206)	29	<0.0001
Total leukocyte count, x 10^9^/L	6.8 (2.5-18.5)	7.8 (3.1-16.4)	40	0.0045
Neutrophil count, x 10^9^/L	3.5 (1.2-10.4)	4.0 (1.4-10.7)	40	NS
Lymphocyte count, x 10^9^/L	0.9 (0.2-4.2)	2.1 (0.2-5.8)	40	<0.0001
NLCR	4.0 (0.9-17.0)	2.1 (0.5-20.3)	40	0.0011
**Non-severe *P. falciparum***				
CRP, mg/L	96 (5–284)	14 (1–250)	116	<0.0001
Total leukocyte count, x 10^9^/L	4.8 (1.3-13.4)	5.6 (2.2-16.9)	161	<0.0001
Neutrophil count, x 10^9^/L	3.0 (0.6-8.8)	2.5 (0.3-11.9)	114	0.0075
Lymphocyte count, x 10^9^/L	0.9 (0.2-3.1)	2.0 (0.3-4.5)	114	<0.0001
NLCR	3.3 (0.5-19.0)	1.3 (0.3-11.0)	114	<0.0001

## Discussion

In contrast to studies in bacterial sepsis, where lymphocytopenia and NLCR were found to outperform CRP in predicting the presence of bacteraemia, lymphocyte counts and NLCR did not allow for an accurate discrimination between malaria patients with severe disease and those without. Even though lymphocytopenia and NLCR had good negative predictive values, CRP was found to be a superior marker in back-to-back analyses.

This lack of diagnostic power might partly be due to the fact that neutrophilia, while often marked in patients with bacterial sepsis, is not commonly seen in malaria. Some studies even report neutropenia [1]. In the present study, neutrophil counts were in the lower range of normal. This is reflected in NLCR values that are much lower than those found in patients with bacteraemia [19] and might be an explanation for the dissimilar performance of the NLCR in bacterial sepsis and malaria. Bacterial co-infection was present in 8% of patients with severe malaria, which is consistent with the findings of a large cohort study on patients with severe malaria admitted to the ICU [20]. However, none of the patients in the present study had a positive blood culture. A major confounding effect of concomitant bacterial infections is, therefore, unlikely. Moreover, regarding the design of the study on NLCR in bacterial sepsis by de Jager *et al.*[19], an important difference with the present study has to be taken into account. In the former study, lymphocytopenia and the NLCR were evaluated as predictors of the presence of bacteraemia in patients with suspected community-acquired bacteraemia, while in the present study these biomarkers were evaluated in a patient group with confirmed malaria. Considering this, the correlation of NLCR with peripheral parasite count and the fact that lymphocyte count increases and NLCR decreases after complete parasite clearance are interesting findings; although NLCR lacks diagnostic power to accurately identify patients with severe malaria, a high parasite load does seem to result in relative lymphocytopenia.

Surprisingly for such a common phenomenon [3-7,21] the mechanism behind malaria-associated lymphopenia has still not been satisfactorily elucidated and remains the subject of debate [22]. The rapid re-emergence of lymphocytes in the peripheral circulation following initiation of treatment has led some authors to suggest transient sequestration during malaria to be responsible [4,5]. The relatively large drop in peripheral lymphocyte numbers would suggest this to be a non-specific effect, *e.g.* pooling in the enlarged spleens of patients [5] rather than a response by malaria-specific lymphocytes only. Others have pointed to the increased propensity of lymphocytes from malaria patients to undergo spontaneous apoptosis *in vitro*[23,24], possibly induced by soluble Fas ligand (sFasL)-Fas interaction [25]. Interestingly, increased apoptosis is also seen in healthy donors from endemic areas, be it to a lesser extent [23,24,26], suggesting chronic stimulation of lymphocytes by environmental micro-organisms may be contributing through activation-induced cell death. Presumably both mechanisms are at work in tandem, with activated lymphocytes sequestering during malaria and rates of apoptosis, whether spontaneous or activation-induced, rising due to infection and peaking following treatment in order to restore homeostasis [27].

In conclusion, the NLCR was found to correlate with parasitaemia, but both lymphocytopenia and the NLCR were inferior to CRP as markers of severe disease in patients with imported malaria in direct back-to-back comparisons. Although these parameters may have proven their usefulness in predicting bacteraemia, they are apparently not useful as predictive markers of severe disease in imported malaria in the acute care setting.

## Competing interests

The authors declare that they have no competing interests.

## Authors’ contributions

MEvW contributed to data acquisition and analysis and writing of the manuscript. KV, MdeMM, PCW, MBM and JvH participated in data analysis and revising the manuscript. RK was responsible for collection of patient data and database management. PJvG participated in study design, data analysis and writing and revising the manuscript. All authors have approved the final version of the manuscript.
